# Reduced Indocyanine Green Clearance Is Associated with Enteral Feeding Intolerance in Septic Patients Without Overt Liver Injury

**DOI:** 10.3390/jcm15124820

**Published:** 2026-06-21

**Authors:** Yingying Hao, Ming Yan, Rujing Bai, Chenyu Li, Chen Qu, Zhuxi Yu, Wenkui Yu, Ning Liu, Tao Gao, Ying Xu

**Affiliations:** 1 Department of Intensive Care Unit, Nanjing Drum Tower Hospital, The Affiliated Hospital of Nanjing University Medical School, Nanjing 210008, China; 2 Department of Intensive Care Unit, Nanjing Drum Tower Hospital, The Affiliated Hospital of Nanjing Medical University, Nanjing 210008, China; 3 Geriatric Medicine Department, The Second Affiliated Hospital of Nanjing Medical University, Nanjing 210011, China

**Keywords:** sepsis, indocyanine green, enteral feeding intolerance, portal vein, gut–liver axis

## Abstract

**Background/Objectives**: The gut–liver axis is central to sepsis, but assessing mesenteric perfusion remains challenging. Indocyanine green (ICG) clearance reflects hepatic blood flow. Since portal flow is derived from mesenteric circulation and supplies most of the liver, reduced ICG clearance may indicate mesenteric hypoperfusion, which can lead to enteral feeding intolerance (EFI). This study examines whether reduced ICG clearance in septic patients without overt liver injury is associated with EFI. **Methods**: This study is a secondary analysis of a prospective cohort study (March–May 2024, 20-bed ICU). Septic patients without sepsis-related liver injury or recent abdominal surgery were included. ICG plasma disappearance rate (ICG-PDR) was measured at admission; patients were grouped by ICG-PDR (≤18%/min vs. >18%/min). The primary outcome was EFI within 7 days. Multivariate logistic regression and correlation analyses were performed. **Results**: Among 77 patients (44 with ICG-PDR > 18%/min, 33 with ≤18%/min), the decreased ICG-PDR group had higher SOFA scores (8.4 ± 4.2 vs. 5.4 ± 3.5, *p* = 0.001) and higher EFI rates (66.7% vs. 43.1%, *p* = 0.041). Univariate analysis showed ICG-PDR ≤ 18%/min associated with EFI (OR = 2.632, *p* = 0.043), but this was attenuated after SOFA adjustment (OR = 2.247, *p* = 0.171). Reduced ICG-PDR correlated with central venous pressure (CVP) (r = 0.626, *p* < 0.001) but not with mean arterial pressure (r = −0.175, *p* = 0.129). **Conclusions**: In septic patients with preserved hepatocyte function, reduced ICG clearance is associated with EFI, but this relationship is largely explained by disease severity (SOFA). Reduced ICG clearance correlates with CVP; however, ICG-PDR cannot distinguish between portal venous and arterial inflow components. The exact mechanism remains speculative.

## 1. Introduction

Sepsis is defined as life-threatening organ dysfunction resulting from a dysregulated host response to infection, and remains a major clinical challenge due to its high morbidity and mortality [1,2]. In its pathophysiology, the liver and gut play pivotal roles: the liver is responsible for clearing pathogens and inflammatory mediators, regulating metabolism, and synthesizing acute-phase proteins [3]; the gut is considered the “motor” of sepsis, where splanchnic hypoperfusion disrupts the intestinal mucosal barrier, promotes bacterial translocation, and amplifies systemic inflammation [4,5]. The close anatomical and functional connection between the gut and liver, known as the gut−liver axis [6,7], underscores the importance of assessing perfusion in both organs to better understand the pathogenesis of sepsis-induced organ dysfunction.

Indocyanine green (ICG) clearance testing is widely used to evaluate hepatic functional reserve. After intravenous injection, ICG is selectively taken up by hepatocytes and excreted into the bile without undergoing enterohepatic circulation [8]. Its clearance rate depends on two key factors: hepatocyte function and effective hepatic blood flow, and can be used to dynamically reflect hepatic excretory function [9]. In clinical practice, some septic patients exhibit a marked reduction in ICG clearance despite having normal transaminase and bilirubin levels. In our recent prospective study, we found that among septic patients without overt liver injury, a decreased ICG-plasma disappearance rate (PDR) (≤18%/min) was associated with higher disease severity scores, elevated lactate levels, and increased mortality [10]. Notably, these patients also showed biochemical evidence of renal impairment. We acknowledge that sepsis-associated renal dysfunction can also be driven by non-perfusion factors such as direct tubular toxicity and inflammatory injury [11]. Nevertheless, what we find particularly meaningful is that reduced ICG clearance is consistent with systemic hypoperfusion, especially in the splanchnic region. This observation suggests that in such patients, impaired ICG clearance is attributable primarily to reduced hepatic blood flow rather than to hepatocyte injury itself.

ICG clearance does not differentiate between portal venous and hepatic arterial blood flow components. Although the portal vein accounts for approximately 70% of total hepatic blood flow and its flow is derived primarily from the mesenteric circulation, the hepatic arterial buffer response may partially compensate for reductions in portal flow, and hepatic oxygen delivery depends largely on arterial supply [12]. Therefore, a reduced ICG-PDR should be interpreted as an indicator of global hepatic perfusion impairment rather than a specific measure of portal venous flow. However, because the portal vein contributes the majority of hepatic blood supply, a reduction in global hepatic perfusion typically suggests that portal venous flow may be decreased. A decrease in portal venous flow implies either increased outflow resistance or reduced inflow from the mesenteric circulation; thus, mesenteric hypoperfusion is likely to coexist with global hepatic perfusion impairment. Mesenteric hypoperfusion can lead to intestinal ischemia, disruption of the mucosal barrier, and motility disturbances, clinically manifesting as enteral feeding intolerance (EFI) [13]. EFI is a common clinical issue in septic patients and is associated with poor outcomes; however, effective bedside tools to assess early mesenteric hypoperfusion are currently lacking.

Against this background, we hypothesized that in septic patients without overt liver injury (not meeting traditional criteria for sepsis-related liver injury), reduced ICG clearance at intensive care unit (ICU) admission is associated with subsequent EFI. This study aimed to investigate this hypothesis.

## 2. Materials and Methods

This study is a secondary analysis of a prospective observational cohort conducted in a 20-bed general adult ICU at the Jiangbei Campus of Nanjing Drum Tower Hospital between March and May 2024. The original study investigated the value of ICG clearance in diagnosing sepsis-related liver injury and predicting mortality [10]. The original study was approved by the Human Research Ethics Committee of Nanjing Drum Tower Hospital (approval No. 2024-119-02) and was conducted in accordance with the Declaration of Helsinki. Written informed consent for data collection and publication was obtained from all patients or their legal representatives. For this secondary analysis, additional patient consent was not required as de-identified data from an existing database were used.

For the current analysis, we included patients from the original cohort who met the following criteria: (1) diagnosed with sepsis or septic shock according to the Sepsis-3 definition [1]; (2) absence of SRLI by conventional criteria (SRLI is defined as meeting at least two of the following: total bilirubin > 34.2 μmol/L, INR [International Normalized Ratio] > 1.5, ALT [alanine aminotransferase]/AST [aspartate aminotransferase] > 2 times the upper limit of normal) [14]; and (3) age ≥ 18 years. Exclusion criteria were: intestinal surgery within 2 weeks prior to ICU admission, chronic liver disease, biliary obstruction, hyperthyroidism, or iodine allergy.

All patients who received enteral nutrition had a nasogastric tube placed for gastric feeding. No patient received post-pyloric feeding during the first 7 days after ICU admission.

Demographic and clinical characteristics, including age, sex, admission diagnosis, and disease severity scores (APACHE II [Acute Physiology and Chronic Health Evaluation II] and SOFA [Sequential Organ Failure Assessment]), were recorded within 24 h of ICU admission as previously described [10]. ICG clearance was measured using a LiMON monitor (PulsioFlex, Munich, Germany). After intravenous injection of 0.25 mg/kg ICG via an antecubital vein, followed by a 10 mL saline flush, ICG-PDR (%/min) was automatically calculated by the device. Based on the previous literature [15] and our prior study [10], an ICG-PDR ≤ 18%/min was defined as decreased clearance.

The following gastrointestinal function outcomes were recorded within the first 7 days after ICU admission:

- Initiation of enteral nutrition (EN): whether EN was started within 72 h of ICU admission;
- Achievement of target EN: whether target caloric intake was achieved within 7 days of ICU admission (based on actual body weight, 25–30 kcal/kg/d);
- EFI: the definitions and prevalence of feeding intolerance vary across studies [16]. In this study, it is defined as the occurrence of any of the following events leading to reduction or interruption of EN [17,18,19]: (1) Vomiting or obvious regurgitation; (2) abdominal distension (increase in abdominal circumference > 3 cm or intra-abdominal pressure > 20 mmHg); (3) diarrhea (>3 loose or watery stools per day, Bristol Stool Form Scale type 6 or 7); and (4) high gastric residual volume (>250 mL on a single measurement). EFI was recorded as a binary variable (yes/no) within the first 7 days after ICU admission.
- Mean Bristol Stool Score: the mean daily Bristol Stool Scale score during the first 7 days.

To control for potential confounding, the following variables were collected:

(1) Hemodynamic parameters (recorded simultaneously with ICG measurement): mean arterial pressure (MAP), central venous pressure (CVP), and heart rate (HR)
(2) Vasopressor use: type and dose were recorded, and the vasoactive–inotropic score (VIS) was calculated [20]: VIS = dopamine (μg/kg/min) + dobutamine (μg/kg/min) + 10 × milrinone (μg/kg/min) + 100 × epinephrine (μg/kg/min) + 100 × norepinephrine (μg/kg/min) + 10,000 × vasopressin (U/kg/min)
(3) Tissue perfusion markers: arterial lactate measured simultaneously with ICG.

Other potential confounders: (1) mechanical ventilation and (2) use of sedatives, analgesics, or neuromuscular blocking agents.

The primary outcome was EFI within 7 days. Secondary outcomes: EN initiation rate within 72 h; EN target achievement rate within 7 days; 28-day and 90-day mortality.

Continuous variables are presented as mean ± SD or median (IQR); and categorical variables as frequencies and percentages. Baseline characteristics and outcomes were compared between groups (ICG-PDR > 18%/min vs. ≤18%/min) using Student’s *t*-test, Mann–Whitney U test, chi-square test or Fisher’s exact test.

Multivariate logistic regression analysis was performed with EFI as the dependent variable and ICG-PDR group as the main independent variable. Based on sensitivity analyses, the final model was adjusted for age and SOFA; lactate was removed because it was normal in both groups and did not change the estimates. Results presented as OR with 95% CI. Correlation between ICG-PDR grouping and CVP/MAP used point-biserial correlation. All statistical analyses were performed using SPSS version 26.0 (IBM Corp., Armonk, NY, USA). Two-tailed *p* < 0.05 was considered statistically significant.

## 3. Results

### 3.1. Baseline Characteristics and Group Comparisons

A total of 77 eligible septic patients were included in this study and divided into two groups based on ICG-PDR: the ICG-PDR > 18%/min group included 44 patients, and the ICG-PDR ≤ 18%/min group included 33 patients. CVP data were available in only a subset of patients (15 in the >18%/min group and 14 in the ≤18%/min group) due to the absence of a central venous catheter.

The prevalence of major comorbidities was similar between the two ICG-PDR groups. Diabetes mellitus was present in 31.8% vs. 33.3% (*p* = 0.888), chronic obstructive pulmonary disease (COPD) in 6.8% vs. 9.1% (*p* = 0.951), pulmonary hypertension in 2.3% vs. 3.0% (*p* = 0.605), heart failure in 11.4% vs. 15.2% (*p* = 0.625), and malignancy in 4.5% vs. 15.2% (*p* = 0.109).

The primary admission diagnoses were pulmonary infection (*n* = 50, 64.9%), urinary tract infection (*n* = 5, 6.5%), abdominal infection (*n* = 3, 3.9%), mediastinal infection (*n* = 3, 3.9%), skin and soft tissue infection (*n* = 2, 2.6%), bloodstream infection (*n* = 2, 2.6%), central nervous system infection (*n* = 2, 2.6%), gastrointestinal infection (*n* = 1, 1.3%), and other sources (*n* = 9, 11.7%).

Among the 36 mechanically ventilated patients, 30 received sedatives, analgesics, or muscle relaxants, and 20 had a central venous catheter; 12 patients with a central line were receiving vasopressors. Additionally, 21 patients (27.3%) met the Berlin criteria for acute respiratory distress syndrome (ARDS) at ICU admission. The proportion was similar between groups (9/44 [20.5%] vs. 12/33 [36.4%], *p* = 0.121).

As shown in Table 1, there was no significant difference in sex distribution. Patients in the ICG-PDR ≤ 18%/min group were older (80.0 vs. 66.0 years, *p* < 0.001) and had higher APACHE II (22.0 ± 7.8 vs. 16.3 ± 6.8, *p* = 0.001) and SOFA (8.4 ± 4.2 vs. 5.4 ± 3.5, *p* = 0.001) scores.

In terms of tissue perfusion and hemodynamics, patients in the ICG-PDR ≤ 18%/min group had significantly higher lactate levels (median 1.7 vs. 1.0 mmol/L, *p* < 0.001)—although both median values were within the normal range—higher CVP (10.2 ± 1.5 mmHg vs. 6.5 ± 2.4 mmHg, *p* < 0.001), higher proportion of septic shock (51.5% vs. 18.2%, *p* < 0.001), and higher VIS (median 4.0 vs. 0.0, *p* = 0.001). There were no significant differences in HR, MAP, mechanical ventilation, or sedative use.

Regarding gastrointestinal outcomes, EN initiation within 72 h was high in both groups (100.0% vs. 93.9%, *p* = 0.352). The decreased ICG-PDR group had a significantly lower rate of achieving target EN within 7 days (63.6% vs. 86.3%, *p* = 0.020) and a significantly higher incidence of EFI (66.6% vs. 43.1%, *p* = 0.041). Among EFI subtypes, high gastric residual volume was more common in the low ICG-PDR group (27.2% vs. 13.6%), while diarrhea occurred in 39.3% vs. 27.2% (*p* > 0.05). Patients in the decreased ICG-PDR group had significantly higher 28-day mortality (39.4% vs. 9.1%, *p* = 0.002) and 90-day mortality (48.5% vs. 18.2%, *p* = 0.004).

### 3.2. Univariate Logistic Regression Analysis for EFI

Univariate analysis (Table 2) showed that APACHE II (OR = 1.086, 95%CI: 1.017–1.159, *p* = 0.014), SOFA (OR = 1.245, 95%CI: 1.082–1.432, *p* = 0.002), and ICG-PDR ≤ 18%/min (OR = 2.632, 95%CI: 1.030–6.723, *p* = 0.043) were associated with EFI. Age, lactate (normal range), MAP, CVP, septic shock, VIS, mechanical ventilation, and sedatives were not significant.

### 3.3. Multivariate Logistic Regression Analysis for EFI

In Model 1 (adjusted for age and lactate), ICG-PDR ≤ 18%/min had borderline significance (OR = 3.047, *p* = 0.054). In Model 2 (adjusted for age and SOFA; lactate removed because it was normal in both groups and sensitivity analysis showed no material change), the association was no longer significant (OR = 2.247, *p* = 0.171), while SOFA score remained independently associated with EFI (OR = 1.230, *p* = 0.007). Age was not significant in either model. As shown in Table 3.

### 3.4. Correlation Between ICG-PDR Grouping and CVP/MAP

ICG-PDR grouping (≤18%/min vs. >18%/min) was significantly positively correlated with CVP (r = 0.626, *p* < 0.001) but not with MAP (r = −0.175, *p* = 0.129).

## 4. Discussion

In this study of septic patients with relatively preserved hepatocyte function, we investigated the association between ICG clearance measured at ICU admission and subsequent EFI. The main findings were as follows: (1) reduced ICG-PDR (≤18%/min) was associated with higher EFI incidence; (2) this association was no longer significant after adjusting for SOFA; and (3) reduced ICG-PDR correlated with CVP but not MAP.

### 4.1. Association Between ICG-PDR and EFI

ICG clearance reflects both hepatocyte function and effective hepatic blood flow. In septic patients without overt liver injury, the finding that reduced ICG-PDR is associated with a higher incidence of EFI therefore suggests that reduced ICG clearance is primarily attributable to decreased hepatic blood flow. The portal vein supplies approximately 70% of hepatic blood flow, and portal blood is derived predominantly from the mesenteric circulation. From a hemodynamic perspective, reduced total hepatic blood flow may imply reduced portal venous flow—particularly in the context of systemic hypoperfusion—which in turn suggests upstream mesenteric hypoperfusion [21]. However, it is important to note that ICG clearance does not differentiate between portal venous and hepatic arterial blood flow. The hepatic arterial buffer response may partially compensate for portal flow reduction, and oxygen delivery is largely dependent on arterial inflow [22]. Therefore, reduced ICG-PDR should be interpreted as an indicator of global hepatic perfusion impairment rather than a specific measure of portal venous flow.

The association between reduced ICG-PDR and EFI was no longer significant after adjustment for the SOFA score. This suggests that the relationship is largely explained by overall disease severity as captured by the SOFA score, rather than by an independent effect of ICG-PDR. Given that patients with reduced ICG-PDR had significantly higher SOFA scores (8.4 ± 4.2 vs. 5.4 ± 3.5, *p* = 0.001) and that SOFA score itself was strongly associated with EFI (OR = 1.229, *p* = 0.009), collinearity between the two is plausible. Larger studies are needed to further distinguish the independent roles of ICG-PDR and SOFA score in identifying patients at risk for EFI.

### 4.2. Correlation Between ICG-PDR and CVP

Another important finding of this study is that reduced ICG-PDR was significantly positively correlated with CVP but not with MAP. However, the mean CVP in the low ICG-PDR group was only 10.2 mmHg, which is mildly elevated and possibly normal for older patients. Thus, this correlation should be interpreted as an association, not as mechanistic evidence of venous congestion.

Physiologically, portal venous flow is influenced by both upstream mesenteric arterial inflow and downstream hepatic venous outflow resistance. Elevated CVP directly increases outflow resistance, thereby reducing effective portal perfusion pressure and decreasing portal venous flow [12]. In septic patients, elevated CVP can arise from multiple factors, including right ventricular dysfunction, fluid overload, intra-abdominal hypertension, or positive-pressure ventilation [23,24,25,26]. In support of this concept, previous work has shown that interventions that raise intra-abdominal pressure (and thus CVP) are associated with reduced ICG-PDR, and that paracentesis (which lowers CVP) improves ICG clearance [27].

Nevertheless, in the present study we did not perform echocardiography, measure BNP, assess right heart function, or separate CVP readings by mechanical ventilation status (20 of 29 CVP measurements were from mechanically ventilated patients, which strongly affects CVP). Therefore, a causal link between CVP and reduced ICG clearance cannot be established from these data. The observed correlation should be viewed as hypothesis-generating, and future studies incorporating echocardiography, BNP monitoring, and invasive or Doppler-derived assessments of venous congestion are needed to clarify the causal relationship.

### 4.3. Comparison with Previous Studies

Our findings are consistent with previous studies demonstrating that ICG clearance reflects hepatic perfusion and predicts outcomes in critically ill patients [6,28,29,30]. In particular, our own prior study in a similar cohort of septic patients without overt liver injury found that reduced ICG-PDR was associated with higher disease severity scores, elevated lactate levels, and increased 28-day and 90-day mortality [10]. However, those previous investigations largely focused on liver dysfunction, hepatic reserve, and overall prognosis, with limited or no exploration of the relationship between ICG clearance and intestinal function. From a different perspective, Tonghui et al. recently demonstrated that gastrointestinal motility disorders can lead to significantly reduced portal vein flow velocity, hepatic arterial blood flow, and hepatic perfusion index, with the degree of motility dysfunction showing a strong negative correlation with hepatic perfusion parameters (r = −0.681, *p* < 0.01) [31]. Their findings provide direct clinical evidence that impaired intestinal function—specifically motility disorders—can compromise hepatic blood perfusion. The current study extends these observations by specifically linking reduced ICG clearance to EFI in septic patients without overt liver injury. To the best of our knowledge, this is the first systematic evaluation of the association between ICG-PDR and EFI in this patient population.

In addition, it is important to acknowledge that patients with COPD, pulmonary hypertension, or heart failure with right heart dysfunction may have chronically elevated CVP [32], which could independently influence ICG clearance and potentially confound the observed association. Similarly, patients with diabetes mellitus may have underlying gastroparesis or motility disorders, and those on chronic opioid therapy may also experience delayed gastric emptying and constipation; these conditions could independently affect enteral feeding tolerance and confound the association we observed [33]. However, in our cohort, the distribution of these comorbidities was not significantly different between the normal and reduced ICG-PDR groups. This similarity suggests that these conditions are unlikely to have substantially confounded the observed association between ICG clearance and EFI. Nevertheless, given the relatively small sample size, we cannot completely exclude the possibility of residual confounding.

This study has several important limitations. First, it is a single-center, secondary analysis with a small sample size (*n* = 77) and no a priori power calculation; findings should be considered hypothesis-generating. Second, CVP data were missing in a substantial proportion of patients (approximately 60%) and could not be included in multivariate models. Third, ICG-PDR does not distinguish between portal venous and hepatic arterial flow; direct measurement of portal flow (e.g., by Doppler ultrasound) was not performed. Fourth, we did not measure intra-abdominal pressure, right ventricular function, BNP, or cumulative fluid balance, all of which could affect CVP and splanchnic perfusion. CVP data were available in only 29 patients, of whom 20 were receiving mechanical ventilation. We did not separate CVP readings by mechanical ventilation status, and mechanical ventilation strongly affects CVP readings. Therefore, the observed correlation may be influenced by positive-pressure ventilation and PEEP. Fifth, the definition of EFI combined upper and lower gastrointestinal symptoms, which may have different mechanisms; the GRV threshold of 250 mL is conservative compared with current practice. Sixth, diarrhea—the most common EFI symptom in our cohort—can be caused by multiple factors unrelated to portal hypoperfusion, such as broad-spectrum antibiotics, Clostridioides difficile infection, or enteral formula composition. We did not systematically document these potential causes, which may confound the association between ICG clearance and EFI. Seventh, comorbidities such as diabetes, chronic opioid use, COPD, pulmonary hypertension, and heart failure were not excluded and may have confounded the results, although their prevalence was low and balanced between groups. Eighth, the lactate difference between groups (1.0 vs. 1.7 mmol/L) was statistically significant, but both values were within the normal range, and adjusting for lactate did not change the results. Ninth, older age may independently reduce ICG-PDR, and the association with EFI was largely explained by SOFA and age. Finally, the study included only septic patients without overt liver injury, so findings may not generalize to those with significant liver impairment.

## 5. Conclusions

In septic patients without overt liver injury, reduced indocyanine green clearance (ICG-PDR ≤ 18%/min) is associated with a higher incidence of enteral feeding intolerance. However, this association is largely explained by overall disease severity as measured by the SOFA score. The positive correlation between reduced ICG-PDR and elevated central venous pressure suggests that venous outflow resistance may contribute to reduced ICG clearance, but ICG-PDR cannot separate portal from arterial components. Bedside ICG clearance testing may serve as a non-invasive indicator of hepatic perfusion, but mechanistic interpretation requires caution. Future prospective studies with larger sample sizes, direct measurement of portal flow, fluid balance, intra-abdominal pressure, and right heart function are needed to validate these findings.

## Figures and Tables

**Table 1:** Baseline characteristics and gastrointestinal outcomes of septic patients stratified by ICG-PDR.

Variables	ICG-PDR > 18%/min (*n* = 44)	ICG-PDR ≤ 18%/min (*n* = 33)	t/u/χ^2^	*p*-Value
Sex			0.0048	0.945
Female, *n* (%)	15 (34.1)	11 (33.3)		
Male, *n* (%)	29 (65.9)	22 (66.7)		
Age, years, Median (IQR)	66.0 (57.0, 77.0)	80.0 (70.5, 83.0)	−3.688	<0.001
APACHEII, score, Mean ± SD	16.3 ± 6.8	22.0 ± 7.8	−3.458	0.001
SOFA, score, Mean ± SD	5.4 ± 3.5	8.4 ± 4.2	−3.464	0.001
Perfusion markers				
Lactate, mmol/L, Median (IQR)	1.0 (0.8, 1.3)	1.7 (1.2, 3.0)	−4.116	<0.001
HR, bpm, Mean ± SD	85.2 ± 19.0	81.0 ± 15.3	1.030	0.306
MAP, mmHg, Mean ± SD	88.5 ± 12.9	83.5 ± 11.6	1.750	0.084
CVP, mmHg, Mean ± SD	6.5 ± 2.4 (*n* = 15)	10.2 ± 1.5 (*n* = 14)	−4.905	<0.001
Septic shock, *n* (%)	8 (18.2)	17 (51.5)	11.810	<0.001
Vasopressor score, Median (IQR)	0.0 (0.0, 0.0)	4.0 (0.0, 15.0)	−3.317	0.001
MV, *n* (%)	19 (43.1)	17 (51.5)	0.526	0.468
Sedatives/analgesics/neuromuscular blockers, *n* (%)	17 (38.6)	16 (48.5)	0.747	0.387
Gastrointestinal function				
EN initiation within 72 h, *n* (%)	44 (100.0)	31 (93.9)	0.866	0.352
Target EN achievement within 7 d, *n* (%)	38 (86.3)	21 (63.6)	5.438	0.020
EFI (any type), *n* (%)	19 (43.1)	22 (66.6)	4.178	0.041
Vomiting	1 (2.2)	1 (3.0)		
Abdominal distension	0 (0.0)	0 (0.0)		
Diarrhea	12 (27.2)	13 (39.3)		
High gastric residual volume	6 (13.6)	9 (27.2)		
Bristol Stool Scale score, *n* (%)			5.489	0.064
1–2, *n* (%)	3 (6.8)	5 (15.2)		
3–4, *n* (%)	15 (34.1)	4 (12.1)		
5–7, *n* (%)	26 (59.1)	24 (72.7)		
28-day death, *n* (%)			10.065	0.002
Survive	40 (90.1)	20 (60.6)		
Death	4 (9.1)	13 (39.4)		
90-day death, *n* (%)			8.071	0.004
Survival	36 (81.8)	17 (51.5)		
Death	8 (18.2)	16 (48.5)		

Data are presented as mean ± SD, median (IQR), or n (%). *p* values were calculated using Student’s *t*-test, Mann–Whitney U test, chi-square test, or Fisher’s exact test, as appropriate. ICG-PDR, indocyanine green plasma disappearance rate; APACHE II, Acute Physiology and Chronic Health Evaluation II; SOFA, Sequential Organ Failure Assessment; HR, heart rate; MAP, mean arterial pressure; CVP, central venous pressure; MV, mechanical ventilation; EN, enteral nutrition; EFI, enteral feeding intolerance.

**Table 2:** Univariate logistic regression analysis for factors associated with EFI.

Variables	OR (95% CI)	*p*-Value
Age, years	0.998 (0.965–1.032)	0.907
APACHEII, score	1.086 (1.017–1.159)	0.014
SOFA, score	1.245 (1.082–1.432)	0.002
Perfusion markers		
Lactate, mmol/L	1.286 (0.878–1.884)	0.196
MAP, mmHg	0.985 (0.950–1.022)	0.428
CVP, mmHg	1.191 (0.884–1.603)	0.250
Septic shock	1.920 (0.720–5.122)	0.193
VIS	1.014 (0.977–1.052)	0.465
MV	1.470 (0.596–3.624)	0.403
Sedatives/analgesics/neuromuscular blockers	2.100 (0.833–5.295)	0.116
ICG-PDR group	2.632 (1.030–6.723)	0.043

Data are presented as odds ratio (OR) with 95% confidence interval (CI). APACHE II, Acute Physiology and Chronic Health Evaluation II; SOFA, Sequential Organ Failure Assessment; MAP, mean arterial pressure; CVP, central venous pressure; VIS, vasoactive–inotropic score; MV, mechanical ventilation; ICG-PDR, indocyanine green clearance plasma disappearance rate.

**Table 3:** Association between ICG-PDR and enteral feeding intolerance: multivariate logistic regression analysis.

Variables	Model 1		Model 2	
	OR (95% CI)	*p*-Value	OR (95% CI)	*p*-Value
ICG-PDR group	3.047 (0.980–9.473)	0.054	2.247 (0.71–7.17)	0.171
Age, years	0.978 (0.941–1.016)	0.907	0.974 (0.94–1.01)	0.200
Lactate, mmol/L	1.135 (0.760–1.695)	0.536	—	—
SOFA, score	—	—	1.230 (1.06–1.43)	0.007

Data are presented as odds ratio (OR) with 95% confidence interval (CI). ICG-PDR, indocyanine green clearance plasma disappearance rate; SOFA, Sequential Organ Failure Assessment.

## Data Availability

De-identified patient data will be available with publication, upon reasonable request to the corresponding author.

## Abbreviations

The following abbreviations are used in this manuscript:

ICG	Indocyanine green
SRLI	Sepsis-related liver injury
PDR	Plasma disappearance rate
EFI	Enteral feeding intolerance
ICU	Intensive care unit
APACHE II	Acute Physiology and Chronic Health Evaluation II
SOFA	Sequential Organ Failure Assessment
EN	Enteral nutrition
MAP	Mean arterial pressure
CVP	Central venous pressure
HR	Heart rate
VIS	Vasoactive–inotropic score
OR	Odds ratios
CI	Confidence intervals
